# Detection of enteric parasite DNA in household and bed dust samples: potential for infection transmission

**DOI:** 10.1186/s13071-020-04012-6

**Published:** 2020-03-18

**Authors:** Rojelio Mejia, Victor Seco-Hidalgo, Diana Garcia-Ramon, Evelyn Calderón, Andrea Lopez, Philip J. Cooper

**Affiliations:** 1 National School of Tropical Medicine, Baylor College of Medicine, Texas, USA; 2 School of Medicine, Universidad Internacional del Ecuador, Quito, Ecuador; 3 Institute of Infection and Immunity, St George’s University of London, London, UK

**Keywords:** Enteric parasites, Soil-transmitted helminths, Protozoa, Transmission, Environment, Dust, Floors, Beds, Ecuador

## Abstract

**Background:**

Enteric parasites are transmitted in households but few studies have sampled inside households for parasites and none have used sensitive molecular methods.

**Methods:**

We collected bed and living room dust samples from households of children participating in a clinical trial of anthelmintic treatment in rural coastal Ecuador. Dust was examined for presence of DNA specific for 11 enteric parasites (*Ascaris lumbricoides*, *Trichuris trichiura*, *Ancylostoma duodenale*, *Necator americanus*, *Strongyloides stercoralis*, *Toxocara canis* and *T. cati*, *Giardia lamblia*, *Blastocystis hominis*, *Cryptosporidium* spp., and *Entamoeba histolytica*) by quantitative PCR (qPCR).

**Results:**

Of the 38 households sampled, 37 had positive dust for at least one parasite and up to 8 parasites were detected in single samples. Positivity was greatest for *B. hominis* (79% of household samples) indicating a high level of environmental fecal contamination. Dust positivity rates for individual pathogens were: *S. stercoralis* (52%), *A. lumbricoides* (39%), *G. lamblia* (39%), *Toxocara* spp. (42%), hookworm (18%) and *T. trichiura* (8%). DNA for *Cryptosporidium* spp. and *E. histolytica* was not detected. Bed dust was more frequently positive than floor samples for all parasites detected. Positivity for *A. lumbricoides* DNA in bed (adjusted OR: 10.0, 95% CI: 2.0–50.1) but not floor dust (adjusted OR: 3.6, 95% CI: 0.3–37.9) was significantly associated with active infections in children.

**Conclusions:**

To our knowledge, this is the first use of qPCR on environmental samples to detect a wide range of enteric pathogen DNA. Our results indicate widespread contamination of households with parasite DNA and raise the possibility that beds, under conditions of overcrowding in a humid tropical setting, may be a source of transmission. 
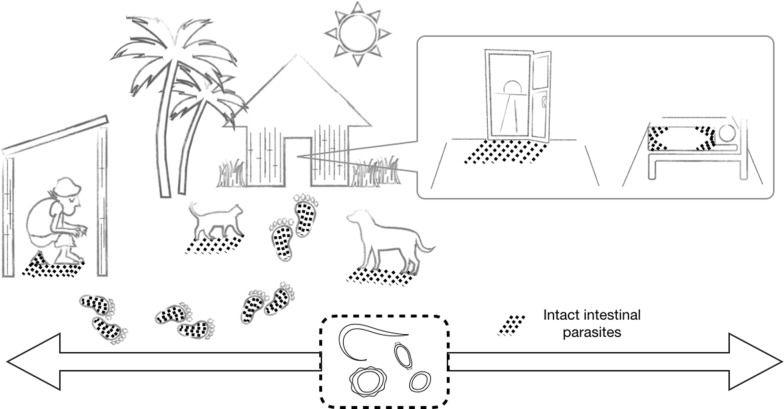

## Background

Intestinal parasite infections are estimated to infect more than a billion humans worldwide and are a major cause of morbidity, especially in children living in conditions of poverty in low- and middle-income countries [1]. Common intestinal parasitic infections of childhood include protozoans (e.g. *Giardia lamblia*) and soil-transmitted helminths (e.g. *Ascaris lumbricoides*). Transmission of intestinal parasites is generally by ingestion or skin contact with infectious parasite stages.

Parasite transmission is considered to occur through contacts with contaminated soil, food and drinking water. Although avoidance of such contacts (e.g. wearing of shoes, access to clean water and sanitation, hand washing after defecation, cleaning of foods, and avoidance of wastewater) [2–4] is considered sufficient to prevent transmission of these infections, studies systematically implementing such strategies (e.g. WASH) have had mixed success [5].

Previous studies have sampled for presence of infectious parasite stages as sources of transmission around households including latrines [6, 7] and within houses [8–10] but none to our knowledge have used sensitive molecular methods to detect specific parasite DNA.

In the present analysis, we analyzed living room floor and bed dust samples for the presence of parasite DNA for a range of fecally-transmitted protozoan and helminth infections to explore the potential for parasite transmission within households in coastal Ecuador. Our data raise the possibility that, under conditions of overcrowding and poor hygiene, intestinal parasitic infections are transmitted through contamination of beds and living room floors with human and animal feces.

## Methods

Dust samples were collected from households of children participating in a cluster-randomized trial comparing bimonthly albendazole (400 mg single dose) with no treatment over a period of 12 months [11]. The trial was done in tropical and sub-tropical communities in Pichincha Province, Ecuador. Sampling was quasi-random at 12 months of follow-up. Dust samples were collected by aspiration from the child’s mattress and the living room floor using an Electrolux vacuum cleaner (1200W) with dust collectors and disposable nylon filters (Indoor Biotech, Charlottesville, VA, USA). Between uses, dust collectors were soaked in acid detergent (2% Citranox, Sigma-Aldrich, St Louis, USA) for 2 h before thorough rinsing with tap water and air-drying. Living room floors and the 4 corners of the child’s mattress were aspirated for 2 min (~ 0.5 m^2^ area). Dust samples were collected in ziplock bags and stored at − 20 °C until analysis. Dust samples were weighed and re-suspended in 35.6% w/w sodium nitrate in water (Alfa Aesar, Heysham, UK) and centrifuged at 1500×*g* for 5 min. Supernatants were filtered through a 3.0 µm SSWP membrane (Millipore, Tullagreen, Ireland) and membranes processed using FastDNA SPIN Kit for Soil (MP Biomedicals, Santa Ana, California, USA) with a heating step of 90 °C for 10 min. Environmental DNA was analyzed by the multi-parallel quantitative polymerase chain reaction (qPCR) as described [12–14] to detect parasite-specific DNA for 11 parasites (*Ascaris lumbricoides*, *Trichuris trichiura*, *Ancylostoma duodenale*, *Necator americanus*, *Strongyloides stercoralis*, *Toxocara canis* and *T. cati*, *Giardia lamblia*, *Blastocystis hominis*, *Cryptosporidium* spp., and *Entamoeba histolytica*)*. Blastocystis hominis*, a species of debatable pathogenicity in humans [15], is a ubiquitous enteric parasite in this population [16] and was included as a marker for fecal contamination of environmental samples. Data from microscopic examination of stool samples by Kato-Katz were available from children at 3 time points during the study (before the start and at 6 and 12 months of follow-up) [11]. Data on potential confounders were collected by maternal questionnaire. Frequencies were compared using Chi-square or Fisher’s exact tests (independent groups) or McNemarʼs test (paired groups). Associations were assessed using Spearmanʼs rank correlation coefficients and measures of effect were estimated using multivariable logistic regression with adjustment for age, sex and household crowding.

## Results

**Fig. 1:**
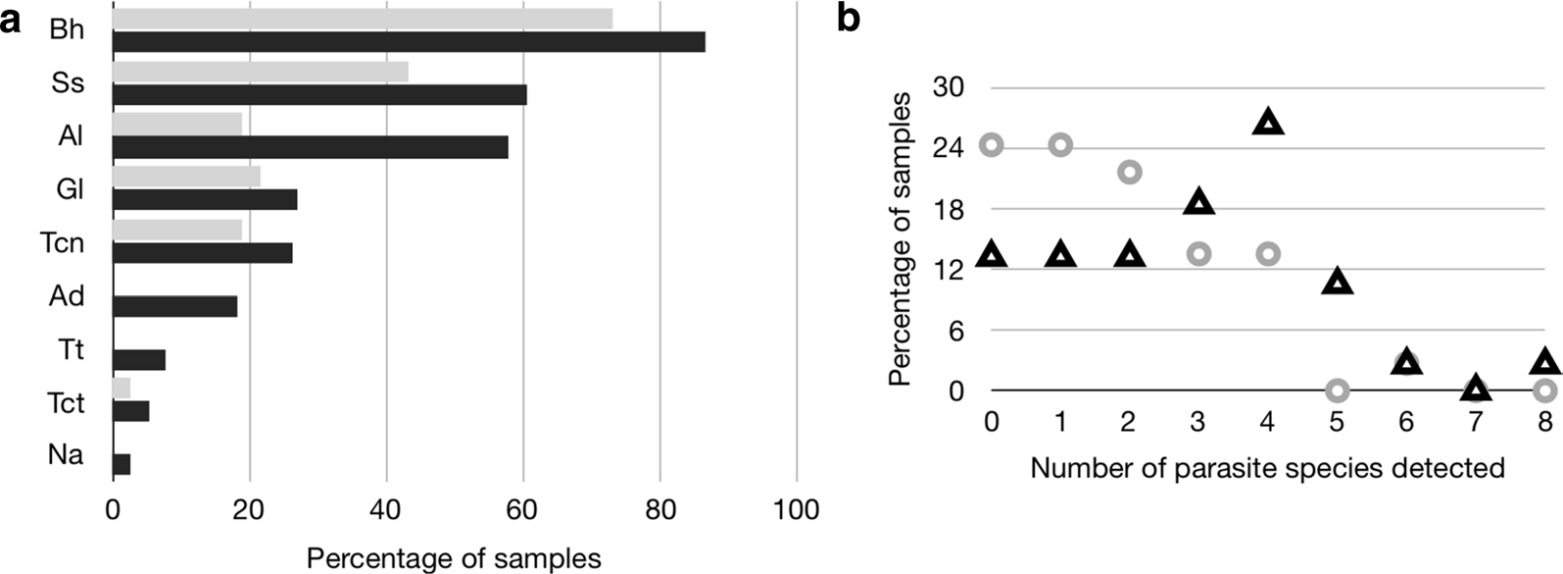
**a** Percentage of dust samples positive for each parasite from living room floors (grey bars) and mattresses (black bars). **b** Percentage of dust samples positive for one or more parasites from living room floors (grey) and mattresses (black). *Abbreviations*: Bh, *B. hominis*; Ss, *S. stercolaris*; Al, *A. lumbricoides*; Gl, *G. lamblia*; Tcn, *T. canis*; Ad, *A. duodenale*; Tt, *T. trichiura*; Tct, *T. cati*; Na, *N. americanus*

**Fig. 2:**
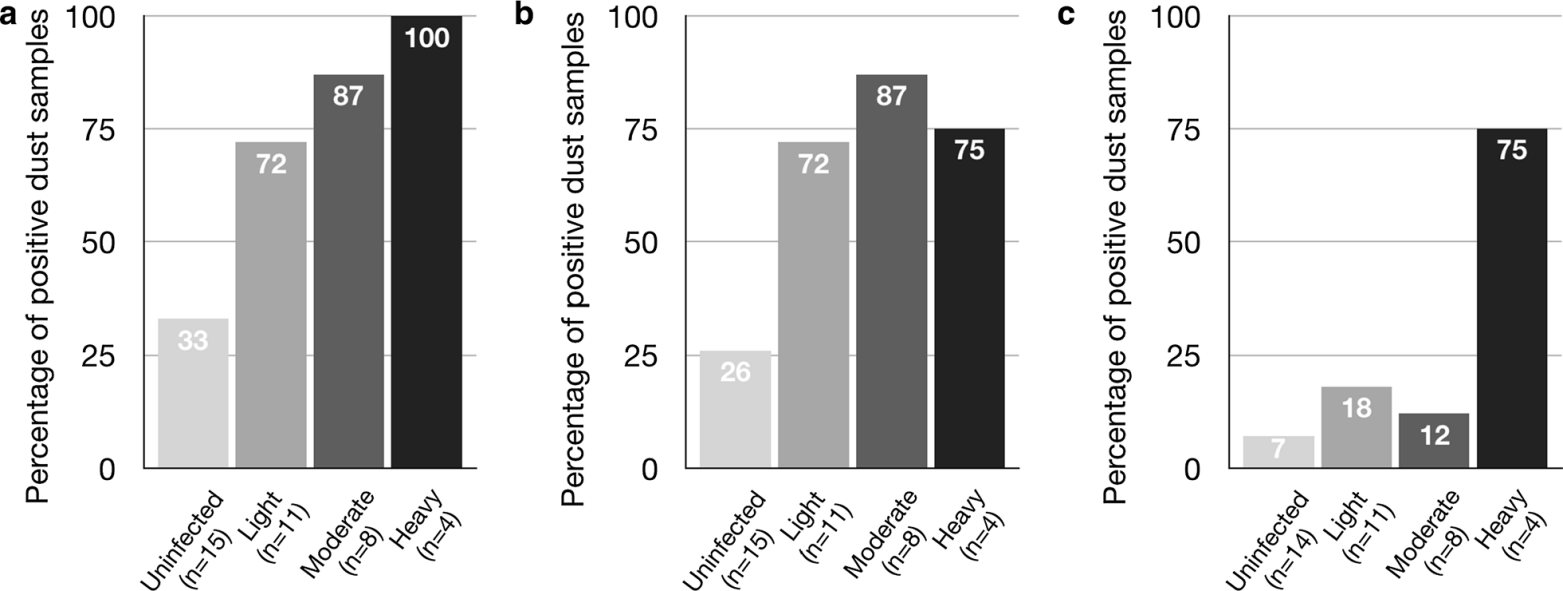
Bar graph showing the percentage of household (all) (**a**), bed (**b**), and living room floor (**c**) dust samples that were positive for *A. lumbricoides* DNA stratified by *A. lumbricoides* infection intensity in children’s fecal samples. Sample numbers for each group are shown (*n*). Intensity groups are those of the WHO (1987) [11]: light, < 5000 eggs per gram (epg); moderate. 5000–50,000 epg; and heavy, > 50,000 epg. Percent positivity for each infection intensity group is shown at the head of each bar

Seventy-five paired dust samples were collected from the houses of 38 children (1 living room floor sample missing). The mean age of the children was 9.7 years (range 7–14 years), 21 (55%) were female, and 22 (58%) attended schools allocated to the anthelmintic treatment arm of the trial. Of the dust samples from the 38 households analyzed, only one house was negative for all parasites tested. Positivity rates by PCR for dust samples were greatest for *B. hominis* (59/75, 79% samples; 37/38, 97% households), followed by *S. stercoralis* (52% samples; 74% households), *A. lumbricoides* (39%; 63%), *G. lamblia* (24%; 39%), *Toxocara* spp. [*T. canis* (23%; 39%); *T. cati* (4%; 8%); any *Toxocara* (24%; 42%)], hookworm [*A. duodenale* (9%; 18%); *N. americanus* (1%; 3%); any hookworm (9%; 18%)] and *T. trichiura* (4%; 8%). No samples were positive for *Cryptosporidium* spp. and *E. histolytica*. Rates of positivity in floor *versus* mattress dust are shown in Fig. 1a: trends of greater rates were observed for all parasites in mattress samples and only mattress samples were positive for hookworms and *T. trichiura*. There was a trend also of more parasites being detected in mattress *versus* floor samples with up to 8 being detected in a single sample (Fig. 1b). Most environmental samples were positive for more than one parasite. Parasites were detected in children’s stool samples by Kato-Katz which is useful only for helminth eggs. Although all children in the treatment arm of the trial were negative for *A. lumbricoides* and hookworm eggs in stool samples by 12 months of follow-up, parasite detection rates in dust samples at this time were similar in the two intervention groups for hookworm (19% for no treatment *vs* 18% for treatment group) and were detectable in dust from intervention households for *A. lumbricoides* (81 *vs* 50%, *P* = 0.049) and *T. trichiura* (13 *vs* 6%), although at reduced frequencies (Table 1). Positivity rates in dust decreased for *A. lumbricoides* and *T. trichiura* but not hookworm among children receiving anthelmintic treatment (curative for *A. lumbricoides* and hookworm) (Table 1). Risk of having a positive bed (adjusted OR: 10.0, 95% CI: 2.0–50.1, *P* = 0.005) but not floor (adjusted OR: 3.6, 95% CI: 0.3–37.9, *P* = 0.280) dust sample for *A. lumbricoides* was significantly associated with having a child with a positive stool sample at baseline. Rates of positivity for *A. lumbricoides* in dust samples increased with increasing infection intensity (Fig. 2); however, only households of children with heavy infection intensities had an elevated risk of a positive floor sample (Fig. 2c), while any infection intensity was associated with an increased risk of positive bed dust (Fig. 2b). Presence of individual parasites in dust was strongly correlated (*P* < 0.05, all comparisons) except for *Toxocara* spp. (data not shown). Bed- and floor sample positivity rates for individual pathogens did not differ except for *A. lumbricoides* (*P* = 0.0007).

**Table 1:** Detection rates for presence of DNA of hookworms, *A. lumbricoides* and *T. trichiura* in dust samples from living room floors and mattresses in households of children receiving (Rx) and not receiving (No Rx) periodic anthelmintic treatments. Proportions of children with positive fecal samples for the same parasites are shown also at the time of dust sampling (i.e. at 12 months)

Parasite	Mattress (%)	Floor (%)	All dust (%)	All dust		Positive stool at 12 months	
	(*n* = 38)	(*n* = 37)	(*n* = 38)	No Rx (*n* = 16)	Rx (*n* = 22)	No Rx (*n* = 14)	Rx (*n* = 17)
Hookworms	18	0	18	19	18	7	0
*A. lumbricoides*	58	19	63	81	50	43	0
*T. trichiura*	8	0	8	13	6	43	6

## Discussion

Enteric parasites are transmitted through ingestion or skin contact with infectious stages in soil, water and food, in a fecally-contaminated environment. Most transmission is considered to occur around or within households [9, 10]. Here, using a sensitive and specific molecular method to detect a range of intestinal parasites, we show for the first time, a potential new source of transmission of these parasites from within the household, especially in bed dust. A previous study from Peru detected *A. lumbricoides* and *T. trichiura* eggs microscopically in living room and kitchen floor samples and also in air samples [8]. In this study, all 9 parasites detected were found more frequently in mattress than floor dust samples indicating greater fecal contamination of beds. Potential sources of infection within households are: (i) living room soil dust is likely to be contaminated by feces brought into the house from defecation sites on feet or footwear of household members (community households used outside toilets shared by one or more families) or by pets; and (ii) mattress dust may be contaminated either by feet/footwear/pets/clothing or deposition from air or direct soiling. The fact that the presence of active infection with *A. lumbricoides* was strongly associated with the presence of *A. lumbricoides* DNA in bed dust (Fig. 2b), independent of positivity in floor dust, implies direct soiling of bedding may be important. Bed dust positivity for *A. lumbricoides* was reduced but not negligible among children receiving periodic albendazole, suggesting that parasite eggs or DNA may persist for long periods in bedding. Finding soil-transmitted helminth (STH) DNA (or eggs) does not necessarily imply the occurrence of transmission, as a period of further development is generally required outside the host (except for rhabditiform larvae of *S. stercoralis*). However, poor bed hygiene, and sharing of beds (a universal practice in this setting) in warm and humid conditions could provide a suitable environment for the development of STH eggs or larvae (as well as of *Toxocara* spp.) to infectious forms, raising the possibility of transmission through contaminated beds. The high prevalence of *S. stercoralis* infection raises the possibility of the sexual stage of the life-cycle occurring in beds. Most protozoan cysts (e.g. *B. hominis*) are probably directly infectious and could immediately re-infect the host or infect other household members when sharing beds. Dogs and cats, generally free to roam around the houses, could directly contaminate beds, e.g. *Toxocara* spp. eggs are frequently present in pet fur [17]. The lack of correlation between presence of *Toxocara* spp. and other enteric parasitic infections may indicate a distinct source of contamination (i.e. direct contamination of beds by pets). This study, from a rural population in tropical Ecuador, provides evidence of almost universal fecal contamination of household dust samples with enteric parasites including bed dust samples and raises the possibility that, in such settings of poverty and overcrowding, beds may provide a focus for the transmission of enteric parasites. Because dust samples were stored frozen, it is not clear if the parasites are viable. During the isolation of parasite stages for subsequent DNA extraction prior to qPCR, we used a 3.0 µm membrane to filter out free DNA and retain eggs and larvae that were separated from organic material in a hypertonic solution. Future studies should sample bed dust for the presence and viability of infectious stages of STH and other enteric parasites and determine if regular cleaning or even the chemical treatment of bed mattresses might reduce transmission.

## Conclusions

To the best of our knowledge, this is the first study to detect enteric parasites in environmental samples using sensitive molecular methods and shows a high level of contamination of dust samples from bed and living room floors with parasite DNA in rural communities in Ecuador. The high levels of contamination of bed dust with parasite DNA that persisted for *A. lumbricoides* after curative treatment raises the possibility that under suitable permissive conditions, bedding may be a source of transmission for enteric parasites.

## Data Availability

Data are available upon request.
